# Seizure and coma secondary to Conn’s syndrome: a case report

**DOI:** 10.1186/s13256-020-02434-5

**Published:** 2020-07-15

**Authors:** Eiman Alseddeeqi, Ajda Altinoz, Najla Ben Ghashir

**Affiliations:** 1 Division of Endocrinology, Sheikh Khalifa Medical City, P.O. Box 51900, Abu Dhabi, United Arab Emirates; 2 Division of General Surgery, Tawam Hospital, Al Ain, Abu Dhabi, United Arab Emirates; 3 Division of Pathology, Sheikh Khalifa Medical City, Abu Dhabi, United Arab Emirates

**Keywords:** Primary hyperaldosteronism, Unilateral adrenal adenoma, Laparoscopic adrenalectomy, Hypertensive encephalopathy, Conn’s syndrome, Case report

## Abstract

**Background:**

Conn’s syndrome is a curable condition if identified properly. It is characterized by autonomous secretion of aldosterone from the adrenal gland cortex. Its morbidity is related to the increased risk of cardiovascular diseases.

**Case presentation:**

We report the case of a 48-year-old man of African descent presenting with generalized tonic-clonic seizure and coma secondary to hypertensive encephalopathy. A biochemical evaluation revealed a very high aldosterone level and an undetectable renin level, both are compatible with primary aldosteronism. The presentation of the following confirms the diagnosis of primary aldosteronism: spontaneous hypokalemia, an undetectable renin level, and a high aldosterone level. Abdominal computed tomography revealed a left adrenal adenoma. Adrenal venous sampling confirmed lateralization of aldosterone excretion from the left adrenal gland. Our patient underwent left laparoscopic adrenalectomy that confirmed a left functional adrenal adenoma. After 12 months of follow up, his hypertension was controlled on only one antihypertensive drug which was down from four drugs preoperatively.

**Conclusion:**

Conn’s syndrome, in this case, was complicated by coma secondary to seizure. Adrenalectomy normalized the hypokalemia and improved resistant hypertension. Potassium supplementation and several antihypertensives were discontinued as our patient became normokalemic and normotensive on one antihypertensive agent.

## Background

Primary aldosteronism (PA) was first described by J. W. Conn in 1955. It was described in the setting of a 4-cm aldosterone-excreting adenoma in association with hypertension, hypokalemia, and excessive urinary excretion of aldosterone [1, 2]. Patients with increased aldosterone secretion have an increased risk of cardiovascular disease attributed to putative causes other than pure hypertension [3]. Conn’s syndrome is considered one of the secondary causes of hypertension in up to 4.8% of cases [4, 5]. In the early stages of the disease, serum potassium might still be within the normal range [6–9]. Normokalemic hypertension is the most frequent phenotype of PA [10].

A hypertensive emergency can present with cerebrovascular manifestations and sequela. The association between neurological manifestations, on the other hand, with PA has been described in the literature [7].

Our case is unusual since it represented the presentation of seizure in the setting of PA which one has to think of as a differential. This case report is of importance to the medical literature as it discusses a severe life-threatening presentation which is curable if addressed properly. Our case manifested with a devastating presentation of neurological complication presenting with seizure and coma. Unilateral adrenalectomy improves resistant hypertension and resolves hypokalemia in patients with Conn’s syndrome [11–16].

## Case presentation

A 48-year-old man of African descent was brought to our emergency department, unresponsive, following an episode of a generalized tonic-clonic seizure. On initial physical examination, he was comatose. Blood pressure revealed a reading of 178/121 mmHg, a heart rate of 105 beats/minute, and a respiratory rate of 18 breaths/minute. His Glasgow Coma Scale was recorded as 3 out of 15, which required intubation and admission to the intensive care unit.

Further history revealed no recreational drugs exposure, no tobacco smoking, and no alcohol consumption; he had no previous history of epilepsy or head trauma. He was not known to have any cardiac comorbidities such as ischemic heart disease or congestive heart failure. His past medical history is remarkable for resistant hypertension; he was on four anti-hypertensive agents but was non-compliant in taking them: atenolol 50 mg, spironolactone 100 mg, amlodipine 10 mg, and valsartan 160 mg. He was first diagnosed as having hypertension 20 years ago. He takes sulfonylurea for type II diabetes mellitus. His family history is remarkable for hypertension in both parents. His family history is negative for the presence of renal diseases, adrenal tumors, or any syndromes associated with adrenal tumors.

He had a body mass index (BMI) of 31. A thyroid examination was consistent with a normal size gland with no nodules and a visual field examination by confrontation to exclude multiple endocrine neoplasia (MEN) syndromes was normal. He did not appear to have cushingoid or acromegaly features.

**Fig. 1:**
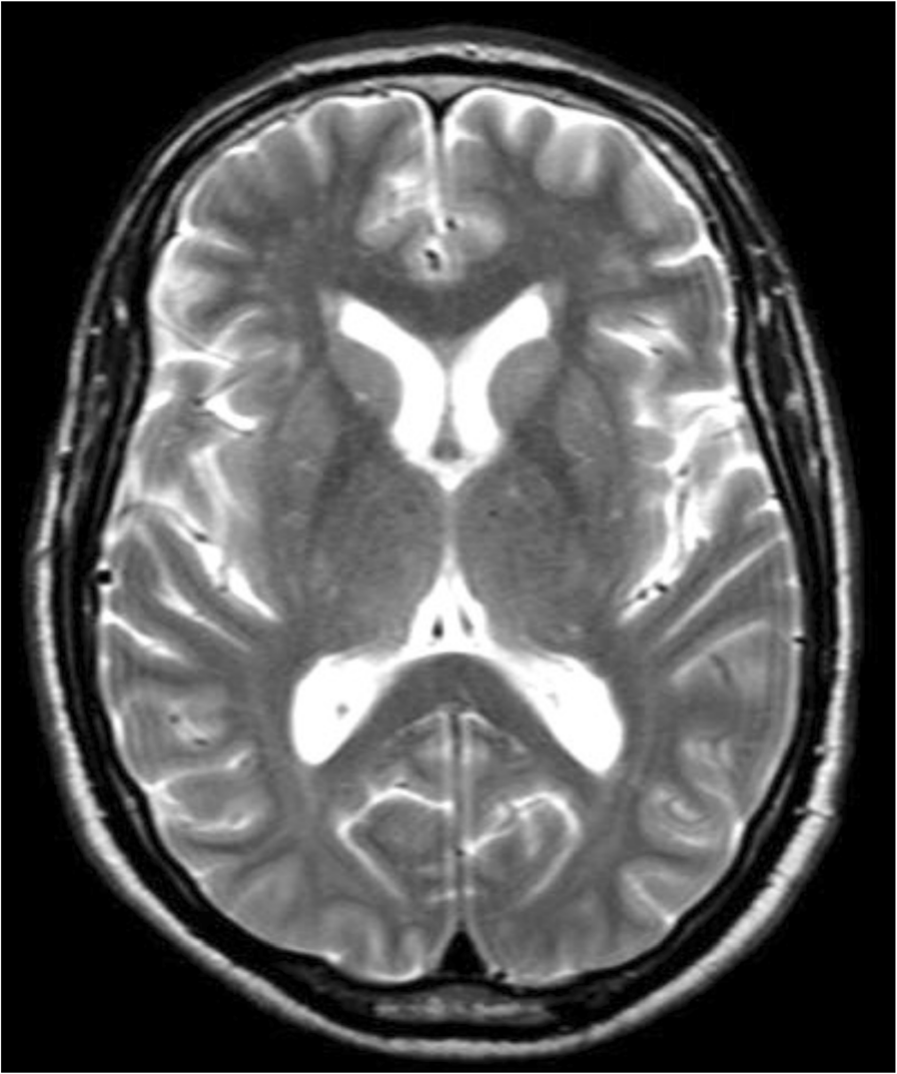
Magnetic resonance imaging of the brain: T2 image, showing no lesions

Initial investigations showed a potassium level of 2.6 mmol/L (normal, 3.6–5.1 mmol/L), a creatinine level of 113 micromole/L, and a blood gas analysis consistent with metabolic alkalosis. His urea level was 5.70 mml/L (normal, 2.8–8.1 mmol/L) and creatinine kinase level read 2.2 mcg/L (normal, < 4.9 mcg/L). Urine analysis was negative for leukocyte esterase and nitrites. A chest X-ray showed no findings of congestive heart failure. A 12-lead electrocardiogram (ECG) showed a sinus rhythm. Troponin-T level was 0.014 mcg/L (normal, < 0.014 mcg/L) and brain natriuretic peptides (BNP) level was 25.9 ng/L (normal, 0–121 ng/L). Calcium level revealed a result of 2.46 mmol/L (normal, 2.23–2.58 mmol/L). A computer tomography (CT) scan showed no brain lesions that could explain the decreased level of consciousness. A magnetic resonance imaging (MRI) of his brain was performed next day and showed no features suggestive of a mass-occupying lesion (Fig. 1). An electroencephalogram was performed and it read normal. Further investigations (Table 1) revealed a potassium level of 2.6 mmol/L (normal, 3.6–5.1 mmol/L) and an aldosterone level of 36.2 ng/dL (normal, 2.8–15.8 ng/dL) with a renin level of less than 0.081 ng/dL (normal, 0.4–2.3 ng/dL). Results of a 24-hour urine analysis ruled out Cushing’s disease and pheochromocytoma (Table 2). Renal ultrasound and cardiac echography results excluded the presence of renovascular and cardiac causes for secondary hypertension.

**Table 1:** Metabolic panel

	Result	Normal Range
Potassium	3.2 mmol/L	3.6–5.1 mmol/L
Creatinine	113 micromole/L	62–106 micromole/L
Aldosterone	36.2 ng/dL	2.8–15.8 ng/dL
Renin	< 0.081 ng/dL	0.4–2.3 ng/dL

**Table 2:** Investigations for pheochromocytoma and Cushing syndrome

	Results	Normal range
Urine dopamine	442 nmol/L	
Urine 24 hours dopamine	1326 nmol/L	< 3240 nmol/L
Urine epinephrine	40 nmol/L	
Urine 24 hours epinephrine	120 nmol/L	< 150 nmol/L
Urine norepinephrine	104 nmol/L	
Urine 24 hours norepinephrine	312 nmol/L	< 570 nmol/L
Urine cortisol	68 nmol/L	
Urine 24 hours cortisol	204 nmol/L	100–379 nmol/L
Urine metanephrine	105 nmol/L	
Urine 24 hours metanephrine	313 nmol/L	< 2000 nmol/L
Urine creatinine	4.68 mmol/L	3.45–2290 mmol/L
Urine 24 hours creatinine	14.04 mmol/L	9–21 mmol/L

Labetalol was initiated in the intensive care unit which controlled our patient’s severe hypertension. He was extubated 2 days later and transferred to a regular care unit sustaining no neurological deficit. Blood pressure was controlled at 122/83 mmHg on amlodipine 10 mg daily, valsartan 160 mg daily, spironolactone 100 mg daily, and metoprolol 100 mg twice daily. Potassium and magnesium required replacement to maintain levels within the normal range.

**Fig. 2:**
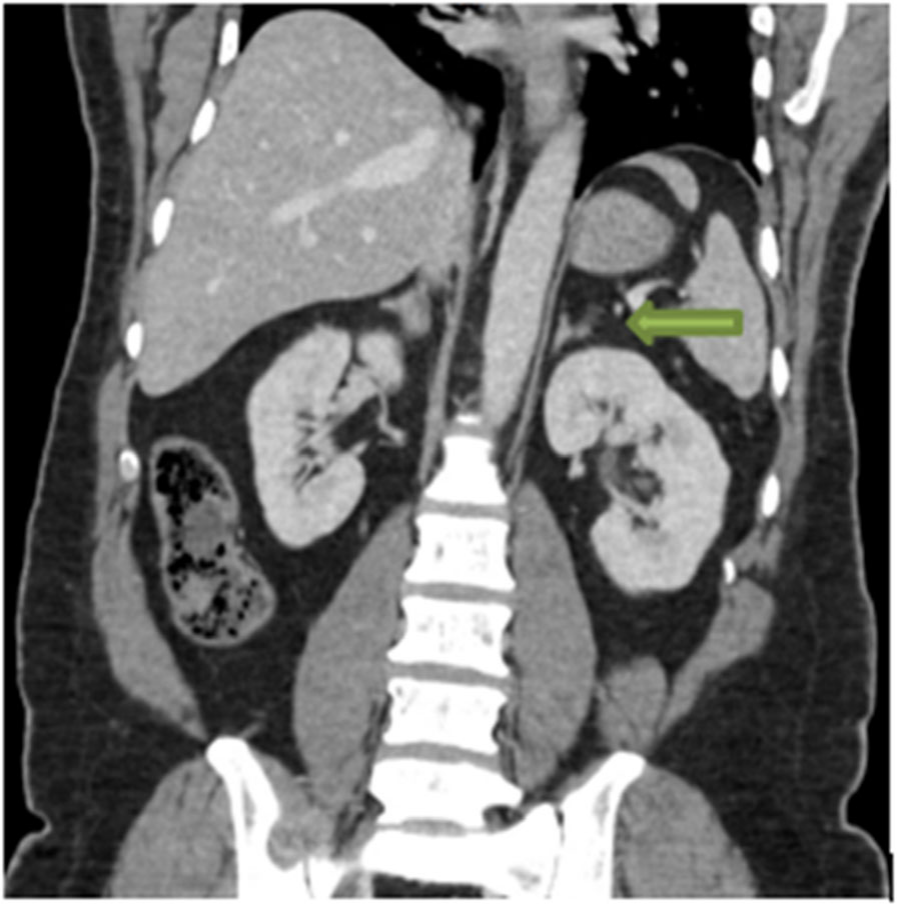
Computed tomography scan of the abdomen showing left adrenal adenoma by the *green arrow*

An abdominal CT (Fig. 2) revealed a well-defined left adrenal gland lesion with an absolute washout time of 85% and a relative washout time of 50%. The lesion measured 12 × 16 × 15 mm in its largest dimensions.

He stayed in our hospital until time of surgery because he lived far away from the hospital and because he was at high risk for uncontrolled severe hypertension. He needed to come off spironolactone for 4 weeks before we performed case detection tests. His potassium level was normalized afterward at 3.9 mmol/L (normal, 3.6–5.1 mmol/L), before performing another set of tests. His aldosterone level remained at 35.3 ng/dl (normal, 2.8–15.8 ng/dL) and renin measured less than 0.08 ng/dl (normal, 0.4–2.3 ng/dL). Aldosterone to renin ratio (ARR) result was consistent with a level above 40 ng/dL per ng/mL per hour (or 3.1 pmol/L per minute). A saline load confirmatory test was not performed as he presented with the following: spontaneous hypokalemia, plasma renin level measuring below detection levels, and a plasma aldosterone level above 20 ng/dL.

Selective simultaneous non-stimulated adrenal venous sampling was performed and it lateralized the lesion to the left adrenal (Table 3). Adrenal vein (AV) to inferior vena cava (IVC) cortisol ratio on both sides was 19 and 15 for left and right AV, respectively (diagnostic cut-off > 5:1), which indicated successful catheterization. Aldosterone level in the left AV was 28,346 nmol/L compared to the right AV, which was 3070 nmol/L. Aldosterone to cortisol ratio of the left AV was significantly higher than that of the right one and it measured 8.7:1 (diagnostic cut-off is > 4:1).

**Table 3:** Selective adrenal venous sampling

Sample	Aldosterone nmol/dl	Cortisol mmol/dl	Adrenal:IVC cortisol ratio	Cortisol corrected aldosterone ratio
Lt. AV	28,346	10,825	19.2	2.61
Rt. AV	3070	8658	15.3	0.3
IVC	644	564		

• To confirm successful catheterization; the adrenal vein cortisol-to-inferior vena cava cortisol ratio’s cut off is greater than a ratio of 5:1. In this case, the ratio is 19 and 15 for left and right adrenal vein, respectively.

• A cut-off for the cortisol corrected aldosterone ratio from high side to low side of more than 4:1 is indicative of the lateralization. In this case, the ratio is 8.7:1

*AV* adrenal vein, *IVC* inferior vena cava, *Lt.* left, *Rt*. right

His blood pressure was controlled on atenolol 50 mg, amlodipine 10 mg, and valsartan 160 mg once a day and potassium supplementation while waiting for surgery. He underwent a unilateral left laparoscopic adrenalectomy.

**Fig. 3:**
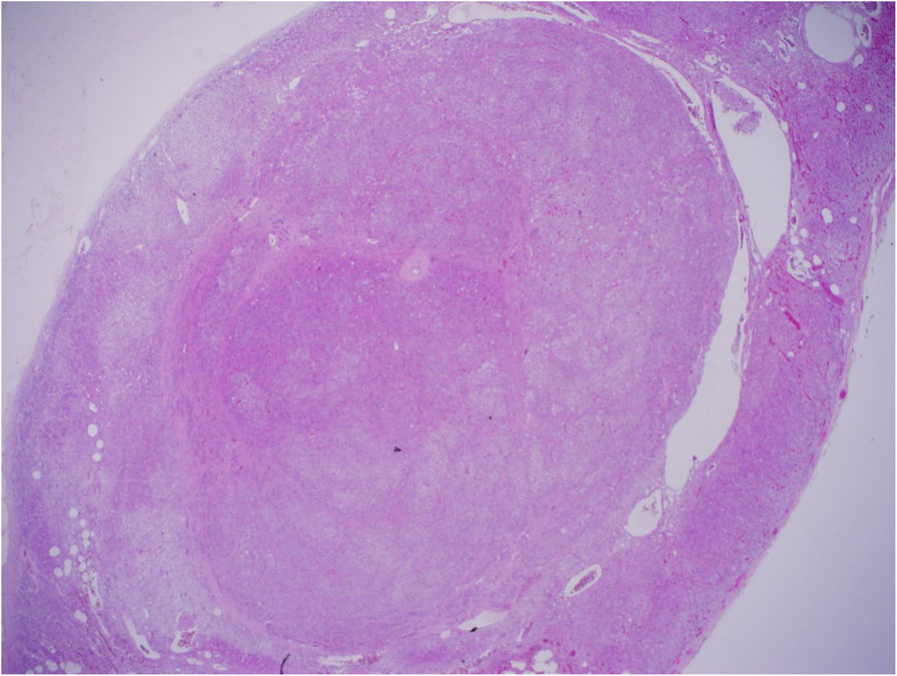
A histology slide with hematoxylin and eosin stain at × 10 power magnification. The adrenal gland adenoma is seen as a circumscribed nodule at the center of the field

A histopathologic examination of the adrenal gland showed an enlarged adrenal gland weighing 20 g, with a discrete, bright golden yellow, ovoid nodule measuring 1 × 1 cm across. Light microscopic examination demonstrated a demarcated adenomatous nodule with a compressed pseudo-capsule, comprising a proliferation of zona glomerulosa-like cells arranged in small nests and cords (Fig. 3).

His postoperative period was uneventful; his blood pressure reading recorded 140/87 mmHg while on three anti-hypertensive agents and his potassium level was 3.9 mmol/L (normal, 3.6–5.1 mmol/L) on no supplementations. At 3-month follow up, his blood pressure read 135/88 mmHg, while being on three anti-hypertensive agents (atenolol 50 mg, amlodipine 10 mg, and valsartan 160 mg), and his potassium level was at 3.8 mmol (normal, 3.6–5.1 mmol) without being on supplements. Baseline aldosterone and renin levels were 13.2 ng/dL (normal, 2.8–15.8 ng/dL) and 0.9 ng/dL (normal, 0.4–2.3 ng/dL), respectively. At 12 months follow up, his blood pressure was recorded as 123/86 mmHg while being on only one anti-hypertensive agent: amlodipine 10 mg daily. Potassium normalized at 4.1 mmol/L (normal, 3.6–5.1 mmol/L) while on no supplementation. Aldosterone and renin levels were at 11.4 ng/dl (normal, 2.8–15.8 ng/dL) and 0.8 ng/dl (normal, 0.4–2.3 ng/dL), respectively.

**Fig. 4:**
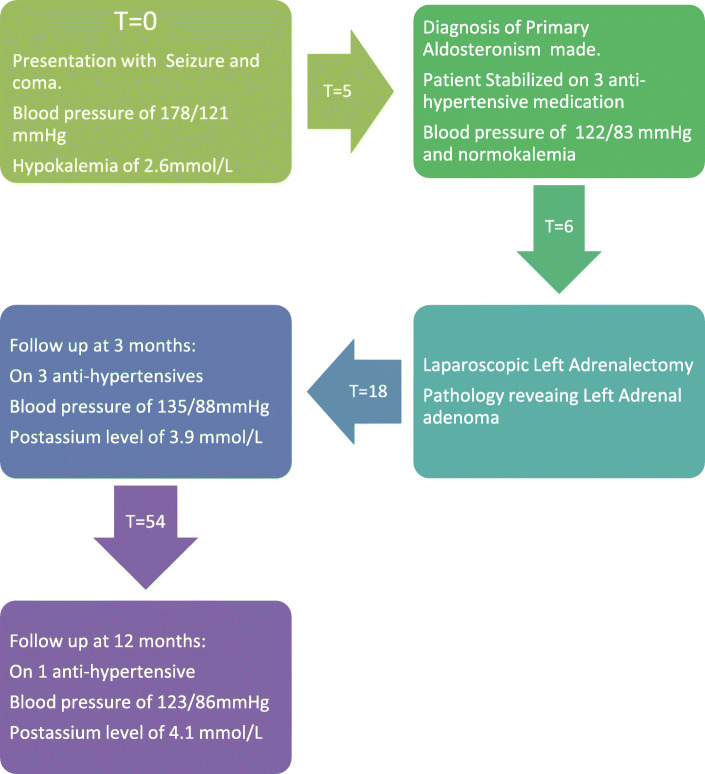
Timeline of events where numerical data identifies time in weeks

Summarizing his postoperative course: a 3-month visit showed a reduction in blood pressure and normalization of potassium level while on three antihypertensives. At 12-month visit, his blood pressure was controlled on only amlodipine 10 mg once daily with normalization of potassium level. A timeline of events is shown in Fig. 4.

Of note, there were no financial, language, or cultural challenges faced in this case.

## Discussion

We report a case of PA due to unilateral adrenal adenoma that was complicated by seizure and coma secondary to hypertensive encephalopathy. To the best of our knowledge, this is the first case reporting a patient presenting with such severe neurological morbidity requiring intensive care management. The usual presentation for PA is resistant hypertension.

Several case reports demonstrate the magnitude of the complications sustained by primary hyperaldosteronism. The most severe complications reported were encephalopathy presenting with headache and resulting in cerebrovascular accidents, and cardiac-related complications manifested by congestive heart failure. Our case, to the best of our knowledge, is the first one describing a presentation of seizure and coma as a result of severe elevation in blood pressure [17, 18]. Miyaji *et al.* reported that out of 427 patients presenting with cerebrovascular accidents, 17 patients had confirmed PA [19]; that category was either an ischemic stroke, intracerebral hemorrhage, subarachnoid hemorrhage, or a transient ischemic attack [19] but none of them presented with seizure or coma.

The differential diagnoses for coma and seizure in our patient were excluded by detailed brain imaging. Cardiovascular risk factors leading to neurological manifestations, such as atrial fibrillation, were ruled out by a normal ECG. Renal failure and uremic encephalopathy were excluded by normal kidney function parameters. Other adrenal pathologies, such as Cushing syndrome and pheochromocytoma were excluded by a 24-hour urine test. Therefore, the coma is explained here by severe acute rise in blood pressure.

Sub-classifying our case, Conn’s syndrome was the etiology for the PA. Conn’s tumor growth is slow, and resulting hypokalemia is similarly of slow progression, leading to adaptations. We, therefore, believe that the encephalopathy was related mainly to the severe sudden elevation of blood pressure rather than hypokalemia which our patient adapted to. Moreover, this adaptation explains the absence of acute clinical signs of hypokalemia and ECG changes. It is important to note that normokalemia is more common than hypokalemia in Conn’s syndrome as the prevalence of hypokalemia is less than 50% [17].

Investigations for hyperaldosteronism should be performed under proper settings; that is, hypokalemia has to be corrected and medications that interfere with ARR results should be stopped for 4 weeks in advance. Verapamil, hydralazine, and doxazosin are the three common drugs that do not interfere with the plasma aldosterone concentration (PAC)/plasma renin activity (PRA) ratio. Beta-blockers and valsartan lower aldosterone level. Being on a beta-blocker or valsartan and sustaining high aldosterone level in this case strongly suggests a primary source of aldosterone secretion [20].

The following biochemical profile confirmed the diagnosis since there was spontaneous hypokalemia, undetectable renin, and a PAC above 20 ng/dL [20–24].

Laparoscopic adrenalectomy is a treatment choice for Conn’s syndrome. Although it effectively resolves the electrolyte imbalance, it might not result in a complete remission of hypertension in the majority of cases, especially in the older age group where there is an underlying component of essential hypertension [25]. An abdominal CT scan revealed an adenoma as a cause for the primary hyperaldosteronism which guided us toward surgery.

Surgery can cure hyperaldosteronism in 60–77% of cases, although it requires around a year or more for hypertension to resolve [26]. The best response to surgical treatment appears to be associated with the presence of an adenoma, an age of under 44 years, duration of hypertension of less than 5 years, and a positive preoperative response to spironolactone [27]. Surgery should prevent our patient from further severe morbidity related to hypertensive emergencies. Our patient fulfilled most of these criteria and he ended up being on one antihypertensive agent, which was needed for an underlying component of essential hypertension given the positive family history. He had partial clinical success with complete biochemical success after unilateral adrenalectomy according to Primary Aldosteronism Surgical Outcome (PASO) study criteria. The PASO criteria is a clinical score used to define response to management of PA. The score is based on six clinical parameters: blood pressure, use of antihypertensive drugs, plasma potassium and aldosterone concentrations, and plasma renin concentrations or PRAs [28].

## Conclusion

We report a severe morbidity related to Conn’s syndrome. Spontaneous hypokalemia and undetectable renin level along with a very high aldosterone level confirm the diagnosis of PA. The potential maximum effect of surgery can commence in up to a year.

## Data Availability

Data sharing not applicable to this article as no datasets were generated or analyzed during the current study.
